# Supplementary material for: Content-rich biological network constructed by mining PubMed abstracts
Source: BMC Bioinformatics. 2004 Oct 8;5:147. doi: 10.1186/1471-2105-5-147 (PMC528731; doi:10.1186/1471-2105-5-147)
Supplement: Additional File 2 — The original results of the above study (non-essential files are deleted to keep the file size under the limit set by BMC bioinformatics). [file 1471-2105-5-147-S2.bz2 › chilibotAdditionalFile2/dip30/312ID11278571E1364/html/ZIPA_FTSZ.html]

 


 **ZIPA** and **FTSZ** 
  
Found 24 abstracts in PubMed, retrieved 24.  
 

 What does Google say? 
 PDF only 
| .edu only 

---

**Interactive relationship** (e.g. stimulation, inhibition, etc)

**Neutral relationship**- Recruitment of  **ZipA**  to the division site by interaction with  **FtsZ** .  Ref: 10209756 Mol Microbiol, 1999
- **FtsZ**   **ZipA**  interaction represents an ancient prototype of the protein protein interaction that enables MAPs to suppress microtubule catastrophe and or to promote rescue.  Ref: 10228152 EMBO J, 1999
- It is suggested that septal ring formation starts by assembly of the  **FtsZ**  ring, after which  **ZipA**  and FtsA join this structure in a mutually independent fashion through direct interactions with the  **FtsZ**  protein.  Ref: 9864327 J Bacteriol, 1999
- **ZipA**  induced bundling of  **FtsZ**  polymers mediated by an interaction between C terminal domains.  Ref: 10960100 J Bacteriol, 2000
- In vitro, wild type  **FtsZ**  interacted with  **ZipA**  with a high affinity in an enzyme linked immunosorbent assay.  Ref: 11278571 J Biol Chem, 2001
- the  **ZipA**   **FtsZ**  interaction is mediated by their C terminal domains.  Ref: 10880432 EMBO J, 2000
- This is the first report of spirals formed by wild type  **FtsZ**  expressed at normal levels and by  **ZipA**  GFP.  Ref: 9696776 J Bacteriol, 1998
- Although a MalE  **ZipA**  fusion binds to  **FtsZ**  filaments, it does not affect the GTPase activity or dynamics of the filaments.  Ref: 10209756 Mol Microbiol, 1999
- It is postulated that the MAP Tau homologous motifs in  **ZipA**  mediate its binding to  **FtsZ** ,.  Ref: 10228152 EMBO J, 1999
- A model is proposed here for how membrane bound  **ZipA**  can reach around the  **FtsZ**  protofilament to bind the carboxy terminal peptide, which faces away from the membrane.  Ref: 11163134 Curr Opin Cell Biol, 2001
- The septal ring in Escherichia coli consists of at least nine essential gene products whose order of assembly resembles a mostly linear dependency pathway FtsA and  **ZipA**  directly bind  **FtsZ**  polymers at the prospective division site, followed by the sequential addition of FtsK, FtsQ, FtsL, FtsW, FtsI, and FtsN.  Ref: 11948172 J Bacteriol, 2002
- I demonstrate that purified  **ZipA**  promotes and stabilizes protofilament assembly of both  **FtsZ**  and FtsZ84 in vitro and cosediments with the protofilaments.  Ref: 10228152 EMBO J, 1999
- In the complex, the 17 residue  **FtsZ**  fragment occupies this entire cavity of  **ZipA**  and binds as an extended beta strand followed by alpha helix.  Ref: 10880432 EMBO J, 2000
- Recruitment of  **ZipA**  to the septal ring of Escherichia coli is dependent on  **FtsZ**  and independent of FtsA.  Ref: 9864327 J Bacteriol, 1999
- Recent data indicate that  **ZipA**  is involved in the assembly of the ring by linking  **FtsZ**  to the cytoplasmic membrane.  Ref: 10880432 EMBO J, 2000
- Two peptide protein interactions are known in this process FtsA and  **ZipA**  bind the C terminus of  **FtsZ** , the bacterial tubulin homologue, which is the first septal component to appear at the septum.  Ref: 11254984 Biochimie, 2001
- The  **FtsZ**  binding FZB domain of  **ZipA**  is significantly larger and encompasses the C terminal 143 residues of  **ZipA** .  Ref: 10960100 J Bacteriol, 2000
- An alanine scanning mutagenesis analysis of the  **FtsZ**  fragment was also performed, which shows that only a small cluster of the buried  **FtsZ**  side chains is critical in binding to  **ZipA** .  Ref: 10880432 EMBO J, 2000
- The results show that  **ZipA**  binds to residues confined to the 20 C terminal amino acids of  **FtsZ** .  Ref: 10960100 J Bacteriol, 2000
- The interaction between  **FtsZ**  and  **ZipA**  occurs through their carboxy terminal domains.  Ref: 10209756 Mol Microbiol, 1999
- In Escherichia coli,  **FtsZ** , a homologue of eukaryotic tubulins, and  **ZipA** , a membrane anchored protein that binds to  **FtsZ** , are two essential components of the septal ring structure that mediates cell division.  Ref: 10880432 EMBO J, 2000
- FtsA and  **ZipA**  localized into a division ring following the same pattern and time course as  **FtsZ** .  Ref: 12754232 J Bacteriol, 2003
- Significantly, we find that the FZB domain of  **ZipA**  is also required and sufficient to induce dramatic bundling of  **FtsZ**  protofilaments in vitro.  Ref: 10960100 J Bacteriol, 2000
- **ZipA** , a membrane protein involved in cell division that colocalizes with  **FtsZ** , was equally dynamic.  Ref: 11854462 Proc Natl Acad Sci U S A, 2002
- **ZipA**  is a MAP Tau homolog and is essential for structural integrity of the cytokinetic  **FtsZ**  ring during bacterial cell division.  Ref: 10228152 EMBO J, 1999
- We have used polymerase chain reaction mediated random mutagenesis in the yeast two hybrid system to analyze this interaction and have identified residues within a highly conserved sequence at the C terminus of  **FtsZ**  as the  **ZipA**  binding site.  Ref: 11278571 J Biol Chem, 2001
- Characterization of  **FtsZ**  residues essential for the interactions with  **ZipA**  and with FtsA.  Ref: 11278571 J Biol Chem, 2001
- Genetic analysis of the Escherichia coli  **FtsZ** . **ZipA**  interaction in the yeast two hybrid system.  Ref: 11278571 J Biol Chem, 2001
- The cell division protein  **ZipA**   [ **ZIPA** ]  has an N terminal transmembrane domain and a C terminal globular domain that binds  **FtsZ** .  Ref: 12107152 J Bacteriol, 2002
- In contrast,  **ZipA**  was capable of binding to all four stable point mutants in the  **FtsZ**  C terminal core but not to the 12 amino acid deletion.  Ref: 10601211 J Bacteriol, 1999
- A C terminal peptide from  **FtsZ**  has been shown to bind  **ZipA**  185 328 in a hydrophobic channel formed by the beta sheet providing insight into the  **ZipA**   **FtsZ**  interaction.  Ref: 10924108 Biochemistry, 2000
- In Escherichia coli,  **FtsZ**  is required for the recruitment of the essential cell division proteins FtsA and  **ZipA**  to the septal ring.  Ref: 10601211 J Bacteriol, 1999
- Structural evidence that the P Q domain of  **ZipA**  is an unstructured, flexible tether between the membrane and the C terminal  **FtsZ**  binding domain.  Ref: 12107152 J Bacteriol, 2002
- These results are consistent with  **ZipA**  acting after Z ring formation, possibly to link the membrane to  **FtsZ**  filaments during invagination of the septum.  Ref: 10209756 Mol Microbiol, 1999
- **ZipA** , an essential component of cell division in Escherichia coli, interacts with the  **FtsZ**  protein at the midcell in one of the initial steps of septum formation.  Ref: 10924108 Biochemistry, 2000
- We have shown that a fusion, which encodes the last 70 residues of  **FtsZ**  in the two hybrid system, is sufficient for the interaction with FtsA and  **ZipA** .  Ref: 11278571 J Biol Chem, 2001
- The bacterial cell division protein  **ZipA**   [ **ZIPA** ]  and its interaction with an  **FtsZ**  fragment revealed by X ray crystallography.  Ref: 10880432 EMBO J, 2000
- **ZipA**  and FtsA are essential division proteins in Escherichia coli that are recruited to the division site by interaction with  **FtsZ** .  Ref: 11847116 EMBO J, 2002
- Furthermore,  **ZipA**  organizes  **FtsZ**  protofilaments into arrays of long bundles or sheets that probably represent the physiological organization of the  **FtsZ**  ring in bacterial cells.  Ref: 10228152 EMBO J, 1999
- **ZipA** , a membrane tethered division protein, binds to a highly conserved short peptide on the carboxyl terminus of  **FtsZ** .  Ref: 11163134 Curr Opin Cell Biol, 2001
- **ZipA**  is an inner membrane protein which is recruited to the nascent septal ring at a very early stage through a direct interaction with  **FtsZ** .  Ref: 10960100 J Bacteriol, 2000
- **ZipA**  is an essential component of the division machinery, and, by binding to both  **FtsZ**  and the cytoplasmic membrane, is likely to be directly involved in the assembly and or function of the  **FtsZ**  ring.  Ref: 9008158 Cell, 1997
- An unexpected similarity between the  **ZipA**  185 328 fold and the split beta alpha beta fold observed in many RNA binding proteins may further our understanding of the critical  **ZipA**   **FtsZ**  interaction.  Ref: 10924108 Biochemistry, 2000

**Non-interactive relationship** (e.g. studied together, co-existance, homology, etc.)

- Concentration and Assembly of the Division Ring Proteins  **FtsZ** , FtsA, and  **ZipA**  during the Escherichia coli Cell Cycle.  Ref: 12754232 J Bacteriol, 2003
- **FtsZ**  and green fluorescent protein GFP fusions to  **FtsZ**  and  **ZipA**  often formed spiral structures in these mutant filaments.  Ref: 9696776 J Bacteriol, 1998
- The recruitment of  **ZipA**  to the septum by  **FtsZ**  is an early, essential step in cell division in Escherichia coli.  Ref: 11278571 J Biol Chem, 2001
- These localization results support the model that E. coli cell division proteins assemble sequentially as a multimeric complex at the division site first  **FtsZ** , then FtsA and  **ZipA**  independently of each other, followed successively by FtsK, FtsQ, FtsL, FtsW, FtsI and FtsN.  Ref: 11703663 Mol Microbiol, 2001
- Consistent with the notion that the ability to bind and bundle  **FtsZ**  polymers is essential to the function of  **ZipA** , we find that  **ZipA**  derivatives lacking an intact FZB domain fail to support cell division in cells depleted for the native protein.  Ref: 10960100 J Bacteriol, 2000
- Here we report that  **FtsZ**  binds directly to a novel integral inner membrane protein in E. coli that we call  **ZipA** .  Ref: 9008158 Cell, 1997
- Direct binding of  **FtsZ**  to  **ZipA** , an essential component of the septal ring structure that mediates cell division in E. coli.  Ref: 9008158 Cell, 1997
- Our results show that  **ZipA**  fails to accumulate in a ring shape in the absence of  **FtsZ** .  Ref: 9864327 J Bacteriol, 1999
- The concentration of the cell division proteins  **FtsZ** , FtsA, and  **ZipA**  and their assembly into a division ring during the Escherichia coli B r K cell cycle have been measured in synchronous cultures obtained by the membrane elution technique.  Ref: 12754232 J Bacteriol, 2003
- Mutations surrounding Asp 373 differentially affected the interactions of  **FtsZ**  with  **ZipA**  and FtsA, indicating that these proteins bind the C terminus of  **FtsZ**  differently.  Ref: 11278571 J Biol Chem, 2001
- Several proteins essential for early steps in cytokinesis, such as  **FtsZ** ,  **ZipA** , and FtsA, were able to localize as bands to potential division sites in pss 93 filaments, indicating that the generation and localization of potential division sites was not grossly affected by the absence of PE.  Ref: 9696776 J Bacteriol, 1998
- Conversely, depletion of  **ZipA**  does not abolish formation of  **FtsZ**  rings but leads to a significant reduction in the number of rings per unit of cell mass.  Ref: 9864327 J Bacteriol, 1999
- The  **FtsZ**  protofilament and attachment of  **ZipA**  structural constraints on the  **FtsZ**  power stroke.  Ref: 11163134 Curr Opin Cell Biol, 2001
- In Escherichia coli, this organelle consists of several essential division proteins, including  **FtsZ** , FtsA, and  **ZipA** .  Ref: 9864327 J Bacteriol, 1999
- In a reverse approach, we use fusions of green fluorescent protein GFP to  **FtsZ** , FtsA and  **ZipA**  to show that these proteins localize to the division site in an FtsL independent fashion.  Ref: 10027987 Mol Microbiol, 1999
- **ZipA**  and FtsA are recruited independently to the  **FtsZ**  cytokinetic ring Z ring and are essential for cell division of Escherichia coli.  Ref: 12634424 Proc Natl Acad Sci U S A, 2003
- MinCD prevented the localization of both  **FtsZ**  GFP and  **ZipA**  GFP, consistent with it preventing Z ring assembly.  Ref: 11673433 J Bacteriol, 2001
- **FtsZ**  and  **ZipA**  are essential components of the septal ring apparatus, which mediates cell division in Escherichia coli.  Ref: 10960100 J Bacteriol, 2000
- In Escherichia coli, at least nine essential gene products participate directly in septum formation FtsA, FtsI, FtsL, FtsK, FtsN, FtsQ, FtsW,  **FtsZ**  and  **ZipA** .  Ref: 11703663 Mol Microbiol, 2001
- In this paper, size exclusion chromatography was used to study the effect of the SulA and MinCD division inhibitors on the oligomerization state of endogenous  **FtsZ**  in cytoplasmic extracts, and immunofluorescence microscopy was used to determine the effect of SulA and MinCD on the formation of  **FtsZ** , FtsA and  **ZipA**  rings at potential division sites.  Ref: 10931335 Mol Microbiol, 2000
- To gain more insight into how the structure is assembled, we studied the interdependence of  **FtsZ** , FtsA, and  **ZipA**  localization using both immunofluorescence and Gfp tagging techniques.  Ref: 9864327 J Bacteriol, 1999
- In contrast, the MinCD division inhibitor did not prevent the oligomerization of  **FtsZ**  in the cell extracts or the formation of  **FtsZ**  and  **ZipA**  ring structures in vivo.  Ref: 10931335 Mol Microbiol, 2000
- We present the X ray crystal structures of the C terminal  **FtsZ**  binding domain of  **ZipA**  and a complex between this domain and a C terminal fragment of  **FtsZ** .  Ref: 10880432 EMBO J, 2000
- Because another proposed function of  **FtsZ**  is the recruitment of FtsA and  **ZipA** , we tested whether the C terminal core domain was important for interactions with these proteins.  Ref: 10601211 J Bacteriol, 1999
- **ZipA**  requires  **FtsZ** , but not FtsA or FtsI, to be localized, indicating that it is recruited by  **FtsZ** .  Ref: 10209756 Mol Microbiol, 1999
- Consistent with this, we found that an  **FtsZ**  mutant unable to interact with both  **ZipA**  and FtsA was unable to assemble into Z rings.  Ref: 11847116 EMBO J, 2002
- The results imply that association of both FtsA and  **ZipA**  with  **FtsZ**  polymers is needed for further maturation of the nascent organelle.  Ref: 11948172 J Bacteriol, 2002
